# Effect of physical activity during COVID-19 on the sleep health of community-level athletes in Australia

**DOI:** 10.1007/s11332-022-00947-8

**Published:** 2022-05-31

**Authors:** Andrew Walsh, Sarah Harris, Philipp Beranek, Shayne Vial, Travis Cruickshank, Mitchell Turner

**Affiliations:** 1 School of Medical and Health Sciences, Edith Cowan University, 270 Joondalup Drive, Joondalup, WA 6027 Australia; 2 Centre for Healthy Ageing, Health Futures Institute, Murdoch University, Murdoch, Australia

**Keywords:** 2019-nCoV, SARS-CoV-2, Novel coronavirus, Athlete(s), Sleep health, SATED

## Abstract

The COVID-19 outbreak presents a serious health challenges, with Australia enforcing tight restrictions, impacting sporting activities and sleep health of many Australians. Routine lifestyle patterns (physical activity and employment) are important to maintaining overall sleep health. Current literature indicates COVID-19 pandemic negatively affected the employment status and sport engagement. The aim of this study was to explore the effect of physical activity during COVID-19 on sleep health, and its association with employment and sport engagement of community-level athletes throughout Australia. Participants self-reported sleep health prior to COVID-19 (pre-sleep) and over the month prior to data collection (during-sleep) using the validated 5-item Satisfaction Alertness Timing Efficiency and Duration questionnaire (SATED). Wilcoxon Signed Rank Test assessed the difference in pre- and during perceived sleep health scores. A generalized linear model was used to assess the impact of sporting and demographic factors on a community athlete’s change in perceived sleep health score. A total of 139 community-level Australian athletes responded. The majority of participants were aged 18–30 and engaged in full-time employment prior to COVID-19 (*n* = 82, 54%). Eight percent of participants were unemployed prior to the COVID-19 pandemic (*n* = 12, 8%). Our findings show that sleep health values were higher during COVID-19, with 91.4% of respondents able to maintain some form of physical activity during the pandemic. Together, our results show better sleep health scores reported by the respondents who maintained or lost employment and maintained sporting engagements during the pandemic.

## Introduction

The emergence of the SARS-CoV-2 virus (COVID-19) along with the strict restrictions implemented in Australia have had a negative influence on the health and well-being on the Australian community [29]. In March 2020, the Australian government implemented rigid restrictions such as the forced closure of sporting facilities, gymnasiums and state borders (Government of Australia 14). The closure of sporting facilities severely impacted community sport athletes, with many sport competitions ceasing or seeing a dramatic reduction in training and playing [13, 31, 32]. Preliminary evidence suggests that the reduced accessibility to organized sports and the uncertainty surrounding COVID-19 has negatively impacted sleep health as restrictions have impacted normal daily living [8, 31]. Facer-Childs et al. [8] found that Australian athletes reported greater sleep latency and later mid-sleep times during lockdown; however, only specific domains of sleep health were measured.

Sleep health is a term used to describe an individual’s sleep, irrespective of any sleep disorder, thus enabling an unbiased examination of sleep, which can be either positively or negatively geared [4]. It includes sleep satisfaction, alertness, timing, efficiency and duration of an individual’s sleep. Superior sleep health is vital for reducing the risk of physical and mental health conditions [9, 12, 15, 18]. Many inventories have been used to assess sleep health [4, 19, 23], with the SATED (sleep satisfaction, alertness, timing, efficiency and duration) questionnaire representing the briefest and most validated measure of sleep health and its respective domains [3, 26, 30, 34].

Many factors can influence sleep health; however, evidence indicates that the disruption of being able to partake in routine physical activity may play a large role in moderating overall sleep health [6, 20, 27]. The COVID-19 pandemic and resultant restrictions have been reported to negatively impact physical activity levels [5, 10, 16, 17]. Studies examining the effect of COVID-19 on the general population show the stress of gaining additional responsibilities for work or maintaining employment has impaired sleep health [5, 33, 34]. However, it is postulated that people who engage in leisure activities, particularly sporting activities, may be able to counterbalance some of the stress associated with loss of employment [1].

Recent evidence in Spanish elite and semi-elite handball players reported an increase sleep duration and poorer sleep quality during the pandemic [22]. An investigation by Facer-Childs et al. [8] found an increase in sleep latency for athletes during lockdown. While informative, these studies examined professional and semi-professional athletes or cast a wide net including anyone who participated in sport. Additionally, only Mon-López et al. [22] quantified changes in participant sleep pre- and during the pandemic isolation period, but they did not account for other domains of sleep health. Finally, professional and semi-professional athletes had a greater reduction in training time [22, 24, 25] but reported better sleep than their non-professional community-based counterparts [5]. To date, there is a paucity of literature on the effect of physical activity during COVID-19 on sleep health in sporting populations, with much of the literature focused on the elite/professional level athletes.

Therefore, the aim of this study was to explore the effect of physical activity during COVID-19 on sleep health, employment and sport engagement of community-level athletes throughout Australia. We hypothesized that community-level athletes would have a reduction in sleep health during the pandemic when compared to pre-COVID levels due to the uncertainty surrounding employment and a reduction in accessing sporting facilities during the pandemic.

## Methods

### Study design

A cross-sectional cohort study design to investigate the sleep health and its association with demographic and lifestyle factors, in Australian community-level athletes. Community-level athletes for this study are defined as anyone participating in a sport at a non-elite level, who do not receive any payment, or do not receive payment as their primary source of income. An online survey was distributed during the initial Australian lockdown period (14th April and 18th May 2020) via word-of-mouth and social media platforms including Twitter, Facebook and LinkedIn. This study was approved by Edith Cowan University Human Resource Ethics Committee (HREC: 2020–01315).

### Participants

Participants were required to be aged 18 years and over, reside within Australia and eligible to participate in a community-level sporting competition in 2020 prior to the COVID-19 restrictions ceasing competition (commenced March 2020). Participants provided informed consent prior to completing the questionnaire. General demographic information was self-reported including sex, age, effect of COVID-19 on employment, and sport participation.

### Sport participation

Participants sport participation was measured through the demographic questionnaire. Participants were asked to select their main sport from a list of sports with one being ‘other’. The participants then reported their current sporting status ranging from professional to community level.

### Employment

Participants employment status was collated through the demographic questionnaire. Participants were asked about their pre-COVID employment status (e.g., full time, part time, casual), their industry of work, if they lost work due to COVID and how much has employment decreased due to COVID.

### Measures

#### Sleep health

Perceived sleep health was self-reported using the validated 5-item Satisfaction Alertness Timing Efficiency and Duration questionnaire (SATED) [3]. Participants reflected on their sleep health for two occasions, prior to COVID-19 (pre-sleep) and over the month prior to data collection (during sleep). Items were scored using a 3-point Likert Scale, 0 (never or very rarely), 1 (sometimes), 2 (often or always) with the total score a sum of the 5-items. Possible total score ranged from 0 to 10 with a lower score indicating poorer sleep health. Previously, a SATED score of < 8 was used as a threshold to indicate poor sleep [7].

### Statistical analysis

Participants were included in analysis if they were community-level athletes, identified as male or female, participated in a team or individual sport, eligible to participate in the 2020 sporting season, and provided complete data for the pre- and during COVID-19-sleep health scales. Additional independent categorical variables included: age group (18–30 years, 31–50 years, > 50 years), ability to continue training (no, yes) and loss of employment during COVID-19 (no change, reduced hours, complete loss). The dependent continuous variable for this study was change in perceived sleep health (SATED—pre- and during COVID-19).

Participant demographic details and change in sleep health scores are described descriptively (Mean [M], Standard Deviation [SD]). As sleep health score distributions were negatively skewed a Wilcoxon Signed Rank Test assessed the difference in pre- and during perceived sleep health scores. A generalized linear model (parameters specified: identity link function, maximum likelihood estimate, Type III model effects and full log-likelihood function), was used to assess the impact of sporting and demographic factors on a community athlete’s change in perceived sleep health score. The model included six perceived confounding factors (comparison category indicated in brackets): state (WA), sex (female), age group (18–30 years), change in employment (no change), type of sport (Individual) and training (continued training). Residuals were visually assessed. SPSS version 27.0 was used for all statistical analysis with significance stated at *p* < 0.05.

## Results

### Participants

A total of 169 participants responded to the survey in April 2020. Seventeen participants were excluded as they did not meet analysis inclusion criteria (professional athlete: *n* = 3; sex non-specified: *n* = 2; unknown sport type: *n* = 2; ineligible to compete in the 2020 season: *n* = 10; incomplete sleep health data *n* = 0). Of the remaining 152 participants, 89 identified as female (59%). Over 90% of participants were amateur level athletes (*n* = 139). Participants ranged in age from 18 to over 71 years, with the majority of participants aged 18–30 years. The majority of participants were engaged in full-time employment prior to COVID-19 (*n* = 82, 54%). Eight percent of participants were unemployed prior to the COVID-19 pandemic (*n* = 12, 8%). Participants were based across all eight states or territories of Australia, with the majority of participants from Western Australia (*n* = 96, 63%), New South Wales (*n* = 18, 12%) or Victoria (*n* = 18, 12%).

### Effect of COVID-19

#### Personal effect of COVID-19

No participants reported being diagnosed with COVID-19, nor knowing someone with confirmed COVID-19. One quarter of participants lost employment due to COVID-19 (*n* = 38), with an additional 18% (*n* = 27) reporting a reduction in employment. Reduced employment ranged from 10 to 90% (*n* = 27, *M* = 49% SD = 29%). Over 40% (*n* = 65) of participant’s financial situation deteriorated due to COVID-19, although conversely 16% perceived their financial situation to improve (*n* = 25).

#### Sport participation

Participants responded across 30 different sports with 22% classified as playing a team sport (*n* = 34) and 78% playing an individual sport (*n* = 118). Common team sports included Hockey and Australian Rules Football, while common individual sports included Triathlon, Tenpin Bowling, Fencing and Cycling.

Ninety-four percent of individual-sport athletes (*n* = 111) were able to still complete some form of training, in comparison to only 82% of team-sport athletes (*n* = 28). Overall, the training of the participants who were able to continue (*n* = 139) decreased by less than 10% up to a reduction of 90%, (*M* = 40%, SD = 30% reduction) compared to pre-COVID-19 restrictions.

### Sleep health

**Fig. 1:**
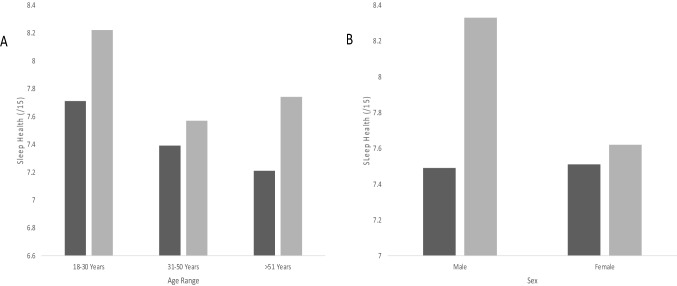
Sleep scores pre- (dark gray) and during (light gray) COVID-19 for age (**A**), sex (**B**)

Sleep health values were marginally higher during COVID-19 (*M* = 7.91, SD = 2.13, Mdn = 8.00), compared to reflections prior to the COVID-19 pandemic (*M* = 7.50, SD = 2.17, Mdn = 8.00). Males had an 8.4% improvement to sleep health values (SD = 1.69) compared to females, with 1.1% improvement during the pandemic (SD = 2.16). While similar prior to the COVID-19 pandemic (Male: 41.3%, *n* = 26; Female: 41.6%, *n* = 37), females reported lower sleep health values (below the threshold < 8 indicating poorer sleep) than males during the pandemic (Male: 25.4%, *n* = 16; Female: 37.1%, *n* = 33, Fig. 1) [7].

**Fig. 2:**
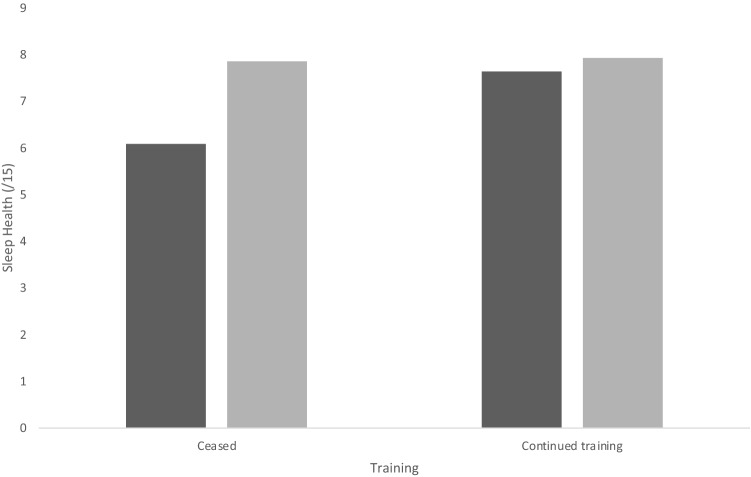
Sleep scores pre- (dark gray) and during (light gray) COVID-19 for training

There was a greater increase in sleep health values for participants who reported cessation in training (*M* = 1.77, SD = 2.09) compared to participants who continued some level of training (*M* = 0.29, SD = 1.96). However, perceived pre-COVID sleep health values were comparatively lower in participants who ceased training, in comparison to participants who continued training (*M*_no training_ = 6.08, SD = 2.87; *M*_trained_ = 7.63, SD = 2.05, Fig. 2).

There was an improvement in sleep health values for participants who lost employment (*M* = 0.84, SD = 2.28) or maintained employment (*M* = 0.36, SD = 1.68). Overall, participants with no change to their employment had marginally greater during-COVID-19 sleep health values (*M* = 8.10, SD = 1.89) in comparison to participants whose employment was affected in some way (*M*_unemployed_ = 7.74, SD = 2.06; *M*_reduction_ = 7.56, SD = 2.89).

Fifty-four participants reported an improved sleep health score whereas 30 participants reported a decrease in sleep health. The remaining 68 participants reported no change. As a univariate model there was a significant median decrease between pre- and during-COVID perceived sleep health scores *z* = 2.88, *p* = 0.004, with a small effect size (*r* = 0.165). When adjusting for confounders, the model indicated sex and ability to train were significantly associated with a change in perceived sleep health (*W*^2^ = 7.37, *p* = 0.007; *W*^2^ = 6.67, *p* = 0.010). In comparison to females, male participants were twice as likely to report improved sleep health (*p* = 0.007, ExpB = 2.32, 95%CI 1.26,4.24). Participants who ceased training were four times more likely to report improved sleep health in comparison to participants who continued to train in any capacity (*p* = 0.010, ExpB = 4.23, 95%CI 1.42, 12.65). All other confounders were not significantly associated with a change in perceived sleep health (State *p* = 0.092; Age *p* = 0.764; Change in employment*p* = 0.282; Sport Type *p* = 0.391).

## Discussion

Emerging literature shows the negative impact of the COVID-19 pandemic and associated restrictions on sleep in the general community and athletic populations around the world [24, 25, 34]. In this study, we explored the effect of physical activity during COVID-19 on the sleep health of Australian community-level athletes. Overall, our findings show that 91.4% of respondents were able to maintain some form of physical activity during the pandemic, which was significantly correlated with sleep health. Our findings also show that sleep health values were higher during COVID-19.

Our finding that participants sleep health improved during the COVID-19 pandemic, compared to pre-COVID-19 reflections. This is contrary with other studies [21, 28] that noted the prevalence of sleep difficulties and insomnia increased [21, 28], greater sleep latency [8] and poorer sleep health [34] during the pandemic. Differences could be due to Australia’s government imposed restrictions, lower incidence and death rates, differences in measures [11, 31]. Previous research from Australia [11, 31], has suggested these measures played a role in better than expected coping and emotional well-being in Australian community-level athletes. Moreover, the continual physical activity evident in our sample (91.45%) may account for improved sleep scores during the pandemic. Interestingly, males reported slightly better sleep health when compared to females during the pandemic (8.4% and 1.1%, respectively). This result confirms previous work [21] which showed that females as well as young people experienced greater sleep disturbances during the COVID-19 pandemic due to greater disturbances to their daily routine [2].

The current findings showed that people who maintained or ceased employment reported better sleep scores than those whose employment was disrupted. These results, in part, support previous findings that show sleep health was positively associated with employment [34]. Minor differences in the results could be explained by the population tested, with Yuksel et al. [34] surveying people from 59 countries, while our study focused on Australian community athletes only. Moreover, participants who experienced a reduction in their employment reported marginally worse sleep health scores. These results are, in part, in contrast to previous findings [5, 33, 34] who found sleep health was impaired for people who maintained work. Whilst unknown, it is plausible that people who had a reduction in employment hours experienced higher employment insecurities than those who did not experience a decrease in hours; however, more research is needed to support this. Therefore, further research is needed to assess the influence of changing employment status had on sleep health during the pandemic.

A vast majority of the participants (91.45%) were able to maintain training through COVID-19 despite the restrictions imposed during the pandemic. A greater increase of sleep health scores was seen for the participants who ceased training during the pandemic. While unknown, it is possible that people who ceased sport specific training still engaged in lockdown approved physical activity, such as running; however, additional investigations are needed to support this supposition. Participants who trained during COVID-19 reported greater mean sleep health scores, contrasting those of Facer-Childs et al. [8] who found that the sleep of Australian athletes declined during the COVID-19 lockdown when compared to their pre-COVID measures. This may be due to 78% of our sample being individual-sport athletes, who had the ability to adhere to social distancing guidelines (e.g., 4 square meters) while training. While Facer-Childs et al. [8] focused predominantly on team-sport athletes who were not able to train or compete during the COVID-19 lockdown period. Our results support that physical activity plays a considerable role in moderating sleep health [20] and it is possible that continued physical activity is important in maintaining global health status, even during a pandemic. Nevertheless, further research is required into the potential buffering effect sport training and participation could have on sleep health during the COVID-19 pandemic.

There are a number of limitations to consider when interpreting our study findings. First, this study had a relatively small sample population from Australia, which may limit the generalisability of study findings. Second, the study asked participants to reflect on their sleep health prior to COVID-19, which can introduce recall bias and therefore the soundness of study findings. Some participants may have received financial support by the Australian Government (e.g., Job-Keeper), which could have reduced stressors associated financial hardship and its impact on sleep health, but this was not measured. Only a small proportion of respondents ceased training completely, affecting generalisability of results. Western Australia was relatively unaffected by COVID restrictions, which may affect generalizability of findings. Data were collected at the start of the pandemic; therefore, the reduction in commitments, activities and understanding of COVID severity and duration may have led to improved initial sleep health. Our findings are in contrast to previous works, primarily that sleep health improved during the pandemic and the rates of physical activity.

## Conclusion

This is one of the first papers that has investigated the effect of physical activity during COVID-19 on sleep health of community-level athletes throughout Australia. Results indicate that higher sleep health scores were associated with people who maintained employment and sporting engagements during COVID-19. While unexpected, sleep health scores were marginally higher during COVID-19 than their pre-COVID-19 reflections. This study helps to shed light on the effects of physical activity during COVID-19 and the subsequent restrictions has on community-level athletes sleep health.
